# Role of Chromatin Architecture in Plant Stress Responses: An Update

**DOI:** 10.3389/fpls.2020.603380

**Published:** 2021-01-12

**Authors:** Sneha Lata Bhadouriya, Sandhya Mehrotra, Mahesh K. Basantani, Gary J. Loake, Rajesh Mehrotra

**Affiliations:** ^1^ Department of Biological Sciences, Birla Institute of Technology and Sciences, Sancoale, India; ^2^ Institute of Bioscience and Technology, Shri Ramswaroop Memorial University, Lucknow, India; ^3^ School of Biological Sciences, Institute of Molecular Plant Sciences, University of Edinburg, Edinburg, United Kingdom

**Keywords:** chromatin remodeling, transcription, nucleosome, histone variants, abiotic stress, epigenetics, intergenerational, transgenerational

## Abstract

Sessile plants possess an assembly of signaling pathways that perceive and transmit environmental signals, ultimately resulting in transcriptional reprogramming. Histone is a key feature of chromatin structure. Numerous histone-modifying proteins act under different environmental stress conditions to help modulate gene expression. DNA methylation and histone modification are crucial for genome reprogramming for tissue-specific gene expression and global gene silencing. Different classes of chromatin remodelers including SWI/SNF, ISWI, INO80, and CHD are reported to act upon chromatin in different organisms, under diverse stresses, to convert chromatin from a transcriptionally inactive to a transcriptionally active state. The architecture of chromatin at a given promoter is crucial for determining the transcriptional readout. Further, the connection between somatic memory and chromatin modifications may suggest a mechanistic basis for a stress memory. Studies have suggested that there is a functional connection between changes in nuclear organization and stress conditions. In this review, we discuss the role of chromatin architecture in different stress responses and the current evidence on somatic, intergenerational, and transgenerational stress memory.

## Plants Utilize Epigenetic and Chromatin-Modifying Strategies to Deal With Stress

Plants utilize highly evolved mechanisms to improve their growth and development to face various biotic and abiotic stresses, in part due to their sessile nature. The plasticity of plants allows them to adapt and survive through these environmental challenges (Gratani, 2014). Chromatin modifications, often associated with alterations in gene expression, have been recognized as significant mechanisms that facilitate plant growth under challenging environments (Fan et al., 2005). The highly condensed and tightly coiled chromatin complex is composed of DNA and histone proteins (Cedar and Bergman, 2009). The tight coiling of chromatin, which is the default state, limits the access of RNA polymerase and other transcription factors to genes. To enable transcription, this compact structure must be opened: this process is termed chromatin remodeling (Bannister and Kouzarides, 2011), and it facilitates the conversion of chromatin from a transcriptionally inactive to a transcriptionally active state. The maintenance of gene activity is controlled by numerous biochemical modifications of chromatin structure, including DNA methylation (Grewal and Moazed, 2003). Some of these modifications can be stably inherited through generations, suggesting that transgenerational adaptation to diverse stresses also has a genetic basis (Pecinka and Scheid, 2012). However, in plants, a limited number of studies have been carried out to validate this transmission of stress-induced changes in chromatin structure. Due to changes in chromatin structure, composition, and location, plants can modify transcription according to changing conditions and can maintain developmental and physiological changes for the long term (Vriet et al., 2015; Perrella et al., 2020). To cope with extreme environmental changes, plants have the power to remember the earlier stress and thus respond more efficiently when they encounter the stress again; this phenomenon is known as priming, which is often related to chromatin modification and may be maintained independently from transcription (Baurle and Trindade, 2020). It is difficult to understand chromatin folding in polyploid plants because polyploidy causes several copies of similar or related genomes in one nucleus. A study was conducted in wheat to understand chromatin architecture, which shows that there are three levels of large-scale spatial organization and concluded that for gene transcription in polyploidy plants, a three-dimensional conformation at multiple scales is the main factor (Concia et al., 2020). The use of high throughput next-generation sequencing (NGS) technologies, well-assembled genome sequences, and the availability of antibodies for a plethora of DNA and histone modifications have all benefited the studies of chromatin remodeling under stresses. This review focuses on the scope and relevance of chromatin architecture in plant stress adaptations.

## Chromatin Remodeling Allows Polymerases, Transcription Factors, and Other Nuclear Proteins to Access DNA

In all eukaryotes, chromatin is packed into nucleosomes; the histone family of proteins makes up a large portion of the chromatin protein component. A nucleosome is a repetitive unit composed of 147 bp of DNA coiled in 1.67 left-handed turns around a histone octamer comprised of pairs of H2A, H2B, H3, and H4 histones (Luger et al., 1997). Histone proteins bear a positive charge and hence can come into close proximity with DNA. H3 and H4 are a part of core histones; they are present on the inside of the nucleosome and are bound to DNA before other histones. Variants of H2A and H2B have been found, which vary in their level of interaction with DNA. Linker DNA is a short strand of a nucleotide sequence that helps in compacting chromatin structure and gene expression regulation (Thoma et al., 1979; Lorch et al., 1999).

When highly condensed, the chromatin architecture prevents access by transcription factors, polymerases, and other nuclear proteins to DNA. Some modifications due to stress signals take place in the chromatin structure, which enables DNA to become accessible. These chromatin remodeling includes shifting or removing histones, introducing histone variants, or posttranslationally modifying existing histones (Eberharter and Becker, 2002).

There are two different strategies among many processes involving two different enzymatic mechanisms to accomplish chromatin organization: One operates through chromatin remodelers that change DNA-histone interactions *via* ATP hydrolysis, and the other utilizes specialized enzymes that methylate DNA or modify histone residues through the addition of covalent modifications (Cedar and Bergman, 2009).

## Chromatin Remodeling Complexes Contain Atpase/Helicase of the SWI2/SNF2 Family Catalytic Core

The SWITCHING DEFECTIVE2/SUCROSE NON-FERMENTING2 (SWI2/SNF2) family of chromatin remodeling complexes (CRCs), part of a large superfamily of helicases and translocases, use the energy obtained from ATP hydrolysis to gain access to DNA sequences (Clapier and Cairns, 2009). The SWI2/SNF2 family CRCs are further subdivided into four classes/subfamilies (Clapier et al., 2017; Ojolo et al., 2018; Table 1).

**Table 1:** The four families of chromatin remodeling proteins and their respective structural domains.

Chromatin remodelers family	Subunits	Domains	References
SWI/SNF (SWItching defective/Sucrose NonFermenting)	BAF, PBAF	HSA, DExx, HELICc, Bromo	Peterson and Workman, 2000
ISWI (Imitation SWItch)	ACF,RSF, CERF, CHRAC, NURF, NoRC, WICH, b-WICH	DExx, HELICc, HAND, SANT, SLIDE	Boyer et al., 2000
CHD (Chromodomain, Helicase, DNA binding)	CHD1, CHD2, CHD3, CHD4, CHD9, NuRD subunits	Chromo, DExx, HELICc	Boyer et al., 2000
INO80 (INOsitol requiring 80)	INO80, Tip60/p400, SRCAP	HSA, DExx, HELICc	Clapier and Cairns, 2009

### SWI/SNF Subfamily Remodelers

The SWI/SNF subfamily remodelers comprise 8–14 subunits initially purified from *Saccharomyces cerevisiae* (Mohrmann and Verrijzer, 2005). A C-terminal bromodomain, a helicase-SANT domain, and a post-HSA domain are present in the catalytic ATPases of most SWI/SNF subfamily remodelers. Homology, dependent on arrangements of SNF2_N and HelicC areas, distinguishes two *Arabidopsis* likely proteins, At5g19310 (CHR23) and At3g06010 (CHR12), and two affirmed proteins, At2g28290 (SPLAYED or SYD) and At2g46020 (BRM), as the nearest homologs of yeast and human SWI/SNF ATPase subunits. BRM and SYD (2193 and 3574 amino acids) represent huge proteins, while CHR12 and CHR23 (1132 and 1054 amino acids) are altogether more modest. AT-hook motifs are present in the C-terminal regions of BRM and SYD, whereas there is no such distinctive C-terminal domain in the CHR12 and CHR23. Decrease in DNA methylation 1 (DDM1) encodes a SWI2/SNF2-like protein, showing that chromatin remodeling is a crucial process for maintenance of DNA methylation (Jeddeloh et al., 1999). In *Arabidopsis thaliana*, DDM1 is one of the important plant epigenetic regulators required for maintaining cytosine methylation in genomic DNA (Dubin et al., 2015). DDM1 is found to enable methylation of DNA bound to the nucleosome. Nucleosomes are prominent barriers to DNA methyltransferases in the absence of remodeling (Lyons and Zilberman, 2017). In *Arabidopsis*, mutations in DDM1 show major methylation losses in all sequence contexts (especially in heterochromatic TEs); small losses can also be seen in genes (Ito et al., 2015). *Arabidopsis* histone H1 inactivation partially rescues the *ddm1* hypomethylation phenotype, showing that DDM1 provides methyltransferase access to H1-containing chromatin (Zemach et al., 2013). A genome-wide reduction in DNA methylation was observed in *ddm1* mutants especially in repeated regions of the genome. *ddm1* mutation induces epigenetic variation, which leads to the steady transmission of morphological phenotypes throughout generations, even if outcrossed from the original mutant backgrounds. Even though the major molecular phenotype of *ddm1* or *met1* mutants is a depletion of DNA methylation, instances of genetic variation as genomic rearrangements, copy number variants (CNVs), and successive DNA transposition have additionally been noticed and may represent a considerable amount of phenotypic variability (Zemach et al., 2013). There are four nonallelic variants of SWI3-type proteins reported in *Arabidopsis* and five in rice. The four *Arabidopsis* variations *AtSWI3A*, *AtSWI3B*, *AtSWI3C*, and *AtSWI3D*, just as their rice partners, all offer the trademark SWIRM (*Swi3p*, *Rsc8p*, and *Moira*), SANT (*Swi3*, *Ada2*, *N-Cor*, and TFIIIB), and Leucine Zipper space with yeast SWI3 and its orthologs in mouse (*Srg3*), *Drosophila* (Moira), and human (BAF170 and BAF155).

In *Arabidopsis*, only BSH (*At3g17590*) shows significant similarity to SNF5 (in yeast), which plays a key role in the organization and functioning of SWI1/SNF1 complexes. The *Arabidopsis* genome encodes two exceptionally comparable homologs of yeast SWP73: *At3g01890* (named *AtSWP73A*) and *At5g14170* (named *AtSWP73B*), which show 83.7% arrangement personality to one another. SWP73 has a functional role in transcriptional activation. The SWI2/SNF2-type ATPase domain belongs to the helicase and NTP-driven nucleic acid translocase superfamily 2 (SF2). This SF2 facilitates interaction with different targeting domains and functional modules, which activates remodeling activities in chromatin structure and thus helps in transcription regulation and DNA repair (Hopfner et al., 2012).

### Imitation Switch Subfamily Remodelers

The Imitation Switch (ISWI) subfamily remodelers comprise of two to four subunits initially purified from *Drosophila melanogaster*. These remodelers consist of plant bromodomains, homeodomains, additional DNA-binding motifs, as well as DNA-binding histone fold motifs (Corona and Tamkun, 2004). In most of the eukaryotes, some specialized proteins form these ISWI family complexes using one or two different catalytic subunits. Nucleosome spacing is optimized by some ISWI family complexes like chromatin-assembly and remodeling factor (ACF) and chromatin-accessibility complex (CHRAC) promoting chromatin assembly and repressing transcription. Whereas certain complexes like nucleosome remodeling factor (NURF) can assist RNAPII activation by randomizing spacing. At the C terminus of the ISWI family, ATPases nucleosome recognition module is formed by a SANT domain (yADA2, ySWI3, hTFIIIB, and hNCoR) adjacent to a SLIDE domain (SANT-like ISWI), which binds to an unmodified histone tail and DNA. The studies on the polytene chromosomes in *Drosophila* larvae suggested the significant impact of ISWI in regulating higher-order chromatin structure.

### Chromodomain Helicase DNA-Binding Subfamily Remodelers

The chromodomain helicase DNA-binding (CHD) subfamily remodelers comprise of 1–10 subunits first purified from *Xenopus laevis*. They vary in their structure due to the diversity in their chromodomains. They can act as transcriptional activators or repressors depending on CHD (Marfella and Imbalzano, 2007). In lower eukaryotes, the catalytic subunit is monomeric; however, in vertebrates, it can be in large complexes. To promote transcription, nucleosomes are ejected or slid by some CHD remodelers whereas some other CHD remodelers have repressive roles like the vertebrate Mi-2/nucleosome remodeling and deacetylase (NuRD) complex [histone deacetylases (HDAC1/2) and methyl CpG-binding domain (MBD) proteins]. CHD1 (identified as a murine protein) interacts with promoter sequences of immunoglobulin and is the founding member of the CHD family. A DNA-binding domain is present at the C-terminal of Chd1 and chd2 proteins that specifically bind to the AT-rich DNA region. The other two proteins CHD3 and CHD4 (a member of the second subfamily) do not contain standard DNA binding domains in their C terminus. However, a pair of PHD Zn-finger-like domain is present at the N-terminal of these proteins. This PHD Zn-finger-like domain is present in several nuclear proteins participating in chromatin-based transcriptional regulation. At C terminus of CHD6 to CHD9 (part of the third subfamily), additional functional motifs like SANT domain or BRK domain are present. There is a discrepancy in the identification of CHD5, as it contains both PHD fingers as well as SANT domain. PHD fingers show interaction with HDAC1 within NuRD in CHD3 and CHD4. CHD remodelers bind with enhancers and help in transcription activation.

### Inositol Requiring 80 Subfamily Remodelers

The inositol requiring 80 (INO80) subfamily initially purified from *S. cerevisiae* is characterized by the presence of a split ATPase subunit with a long insertion found in the middle of the ATPase domain, which binds with the helicase-related (AAA-ATPase) Rvb1/2 proteins and one ARP protein. It is involved in transcription activation and DNA-double-strand break (DSB) repair (Bao and Shen, 2007). Higher orthologs of the INO80 family include hINO80, hSRCAP (SNF2-related CREB-activator protein), and p400, also having HAT activity. CRCs from different subfamilies are involved in diverse plant physiological processes like cell differentiation, meristem establishment, floral morphogenesis, organ development, phytohormone signaling, and biotic and abiotic stress tolerance. RuvB-like helicases, the unique proteins for INO80 and SWR1 complexes, are related to the bacterial RuvB helicase, which takes part in DNA repair. The member of this family binds to the histone variants of H2A: H2A.X and H2A.Z. *In vivo* INO80 complex is involved in nucleosome eviction, while the SWR1 complex catalyzes the replacement of a canonical H2A-H2B dimer with an H2AZ-H2B variant dimer. The ATPase subunits of the INO80 family and other ATPases in the SNF2 helicases are different, as a long spacer region is present in the INO80 complex that splits the conserved ATPase domain. This region binds with RuVB-like subunits and Arps. The helicase-SANT domain (HAS domain) necessary for the binding Arps and actin components is also present in the motor subunits of INO80 protein. The involvement of IN080 complexes in DNA repair is suggested by the presence of RuvB-like helicases.

## Chromatin Modifications in Plant Stress Tolerance

**Figure 1:**
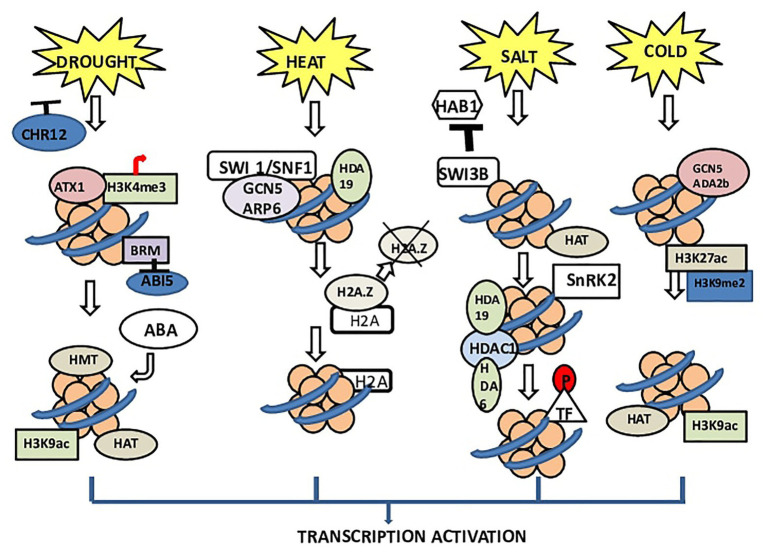
Chromatin architecture under different stresses in plants. BRM (SNF/Brahma), CHROMATIN REMODELING 12 (CHR12) acts as a negative regulator. The receptors of drought stress deactivate CHR12 to promote plant productivity. During stress, BRM activity gets inhibited. BRM has been reported to control ABI5 expression especially by regulating the nucleosomal stability in the promoter and coding regions of this gene. BRM inhibits the expression of ABI5, thus initiating ABA biosynthesis. In heat stress, switching defective/sucrose nonfermenting (SWI1/SNF1) complex interacts with GCN5 and ARP6, which dissociates H2A.Z. The dissociation of H2A.Z causes transcription of downstream genes. Normally, the complex of ARP6 with SWI1/SNF1 plays important role in the insertion of H2A.Z into the nucleosome and replacing H2A. In the case of salinity, the receptors of salt stress inhibit the binding of SWI3B and HAB1. Due to this nonassociation, SNF1-related kinase (SnRK2) remains active, which leads to phosphorylation of transcription factors and finally transcription of genes. Under cold stress, ADA2b, which is a transcriptional activator of HATs, interacts with GCN5 (*Arabidopsis* HAT) and enhances the HAT activity of GCN5. This interaction increases the histone acetylation level.

Plants exploit chromatin modification mechanisms, (i) CRCs and (ii) chromatin-modifying enzymes, to overcome various biotic and abiotic stresses (Asensi-Fabado et al., 2017). In *Arabidopsis*, during stress, RESTRICTED TO NUCLEOLUS 1 (REN1) was found to be incorporated with nucleoli and helps in pollen development (Reňák et al., 2014). STRESS RESPONSE SUPPRESSOR 1 and 2 (STRS1 and 2) are DEAD-box RNA helicases; loss-of-function mutations in these proteins result in plants resistant to various stresses (Kant et al., 2007), whereas overexpressing STRS1 or STRS2 results in stress hypersensitivity. These proteins have a transient interaction with the nucleolus during diverse stress conditions, with different kinetics. RNA-directed DNA methylation (RdDM) pathways can inactivate some genes (Figure 1).

In plants, histone acetyltransferases (HATs) and HDACs catalyzing histone acetylation and deacetylation show a role in cold responses (Kim et al., 2015). In *Arabidopsis*, HISTONE DEACETYLASE 6 (HDA6) is upregulated by cold stress and positively regulates freezing tolerance (Luo et al., 2017). HDACs appear to directly activate maize (dehydration responsive element binding protein 1) DREB1 (ZmDREB1) gene expression and histone hyperacetylation under cold stress (Yu et al., 2018; Ding et al., 2019). According to a recent study to regulate the expression of COR genes (COR47 and COR15A), HOS15 works together with HISTONE DEACETYLASE 2C (HD2C) by directly binding to their promoters (Park et al., 2018; Figure 1).

In *Arabidopsis*, salinity tolerance is determined by expression levels of DEK3 (a DEK domain-containing protein), which acts in association with DNA topoisomerase (Waidmann et al., 2014). Members of the acetylation lowers binding affinity (ALBA) family are expressed in rice plants under drought stress, but their exact mechanism in chromatin organization is not yet evident (Verma et al., 2014). According to a recent study in *A. thaliana* seedlings subjected to four abiotic stresses (heat, cold, salt, and drought), there was no change observed in a large portion of chromatin. Chromatin accessibility was increased in case of extreme temperatures, while the result for chromatin accessibility did not change much in case of drought and salt stresses (Raxwal et al., 2020).

Epigenetic regulators have been found to affect the intranuclear localization of STRSs, hence showing that they have a role to play in the silencing of stress response genes with chromatin alterations (Khan et al., 2014). Sumoylation (attachment of SUMO moiety) is one of the common posttranslational protein modifications in response to several plant stresses (Miller et al., 2013; Elrouby, 2017). During stress, SUMOylation could play an essential part in changing the messenger RNA (mRNA) profile. SUMOylation of RNA binding proteins and elements engaged with 3' pre-mRNA processing, RNA editing, transcription termination, and mRNA export (Richard et al., 2013; Lamoliatte et al., 2014) have assisted with extending the function of this modifier to the field of RNA processing and metabolism (Rouviere et al., 2013). It is found that SUMO pathway enzymes colocalize in nuclear bodies and substructures along with segments of the RNA processing machinery. A few individuals from the protein inhibitor of STAT (PIAS) family of SUMO E3 ligases localized to nuclear speckles, which are subnuclear structures advanced for pre-mRNA splicing factors (Lamond and Spector, 2003; Hall et al., 2006). According to a study, SUMO-1 and the E2-conjugating enzyme ubc9 are localized to Cajal bodies (sites of maturation of snRNPs) necessary for pre-mRNA processing (Navascues et al., 2008). Multiple putative SUMO targets are present in functional capping, splicing, polyadenylation, termination, and mRNA export processes (Richard et al., 2017). During heat stress, SUMOylation has been accounted for controlling DNA methylation patterns, which, along with the stress-up-regulated SUMOylation of *Arabidopsis* variants of histone acetylases/deacetylases, for example, GCN5/ADA2B (Sterner et al., 2006) and HDA19 (To et al., 2011), may then assist in the conversion of euchromatic regions into heterochromatic regions during stress.

The MORC family is a subfamily of microrchidia (MORC) GHKL ATPases (Gyrase, Hsp90, histidine kinase, and MutL) superfamily. MORC protein was initially isolated from mouse, which is important for meiotic nuclear division (Watson et al., 1998). Thereafter, MORC genes have been identified in mammals (Pastor et al., 2014), *Caenorhabditis elegans* (Moissiard et al., 2012), and different plant species, including *Arabidopsis* (Kang et al., 2008), tobacco, barley, and potato. In *Arabidopsis*, seven members of MORC are identified and five members in barley. Microrchidia (MORC) subfamily is highly conserved and comprises widespread domain architectures, which enables it to link with epigenetic regulation and signaling-dependent chromatin remodeling (Lorković, 2012; Li et al., 2013). The role of MORC in chromatin-based transcriptional gene silencing (TGS) is studied in *Arabidopsis* (Lorković, 2012). MORCs interacts with other proteins and derive versatility in chromatin-associated functions. Mutations in two *Arabidopsis* genes, *AtMORC1* and *AtMORC6* (members of conserved MORC ATPase family), show de-repression of DNA-methylated genes and TEs. Enhanced interaction of pericentromeric regions and the genome, decondensation of pericentromeric heterochromatin, and transcriptional defects that are mainly focused on loci residing in pericentromeric regions are noticed in *atmorc1* and *atmorc6* mutants. In eukaryotes, MORC ATPases are proposed to be the conserved regulators of gene silencing (Moissiard et al., 2012). The MORC proteins are a subset of the GHKL ATPase superfamily. These proteins have been described as components involved in plant immunity in *Arabidopsis*. Resistance to *Phytophthora infestans* in solanaceous plants was compromised in silenced *StMORC1* in potato and enhanced in overexpressing lines, indicating that *StMORC1* positively affects immunity, whereas the resistance to *P. infestans* in *SlMORC1* silenced in tomato or *NbMORC1* silenced in *N. benthamiana* was increased. It was also observed that transient expression of *StMORC1* in *N. benthamiana* triggers cell death, initiated by infestin1 (INF1), while *SlMORC1* or *NbMORC1* expression represses it (Manosalva et al., 2015). *Arabidopsis MORC1*, formerly named CRT1 (compromised for recognition of TCV 1), identified as a hereditary screen to recognize components associated with the TCV resistance signaling pathway (Kang et al., 2008). *Arabidopsis* CRT1 is necessary for effector-triggered immunity. CRT1 possesses the ATPase and 5S domains, which is a characteristic of MORC proteins. These proteins are involved in DNA modification and repair (Kang et al., 2012) It has been studied that CRT1 and CRH1 (closest homolog of CRT1) are necessary for basal resistance, pathogen-associated molecular pattern (PAMP)-triggered immunity, systemic acquired resistance, and nonhost resistance. The level of CRT1 in the nucleus increases by PAMP treatment or infection with an avirulent pathogen. In *Arabidopsis*, resistance to Turnip crinkle virus (TCV) is represented by the resistance protein HRT (HR to TCV) and its related avirulence factor, the viral coat protein. Plants not having HRT fail to build up an HR after TCV infection permits systemic viral spread and results in the death of the plant. CRT1 physically interact with HRT and 10 other R proteins; these R proteins are mainly inactive. CRT1 possesses two close and four distant homologs; silencing of the two closest homologs, CRH1 (CRT1 homolog 1) and CRH2, compromised TCV resistance to a far extent in comparison to *crt1. crt1-1* mutation and silencing of CRT1 family members compromise cell death triggered by the R proteins. Reduced resistance to avirulent *Pseudomonas syringae* (Pst) and *Hyaloperonospora arabidopsidis* was observed in double knockout (dKO) in the Col-0 background, *crt1-2 crh1-1*, which lacks CRT1 and its closest homolog. The knockout of CRT1 gene results in severe susceptibility to both virulent and avirulent *H. arabidopsidis*. These results show that CRT1 is a very crucial factor in multiple levels of plant immunity (Kang et al., 2012). GHKL ATPase motif is present in several prokaryotic and eukaryotic proteins; these proteins are involved in heat shock responses (*Hsp90*), rearranging DNA structure (gyrase or topoisomerases), signal transduction (histidine kinase), or DNA mismatch repair (MutL; Iyer et al., 2008). In *Arabidopsis*, nucleosome assembly proteins (NAPs; NRP1 and NRP2) localized in the nucleus, formed protein complexes, and acted as H2A/H2B chaperones. These protein complexes help in the regulation of chromatin organization in epigenetic inheritance, as they specifically bind to histones H2A and H2B (Zhu et al., 2006). NAP1 is evolutionary preserved from yeast to humans. In *Arabidopsis*, these NRP proteins are involved in many biological processes, for example, cell-cycle control, heat tolerance, somatic homologous recombination, DNA repair, root meristem formation, and genome defense under genotoxic stress (Gao et al., 2012). NRP proteins localized predominantly in the nucleus (Gonzalez-Arzola et al., 2017) genetically interact with the SWR1 core components and link with H2A.Z. It is proposed that, in *Arabidopsis*, NRP proteins counteract the activity of the SWR1 complex and associate with the dynamic regulation of H2A.Z (Wang et al., 2020).

Evolutionary conserved SnRK1 kinases (Snf1-RELATED KINASE1) govern metabolic adaptation during low extended darkness by controlling C/S1-bZIP signaling in *A. thaliana* (Pedrotti et al., 2018). Plants face continual environmental fluctuations because of their sessile nature, which may harm their energy storage. Plant SnRK1s adjust metabolic, developmental, and transcriptional processes due to such challenges (Hey et al., 2010; Smeekens et al., 2010). SnRK1s KIN10 and KIN11 handle energy loss by controlling the stress-responsive genes expression and signaling of abscisic acid in *Arabidopsis* (Baena-Gonzalez et al., 2007; Jossier et al., 2009). Calcineurin B-like interacting protein kinase 15 controls rice *OsSnRK1* (Lee et al., 2009) and further derepresses the expression of (glucose) Glc-repressed gene in the embryo (Lu et al., 2007) to modulate early seedling growth and seed germination. During evolution, SNF1/AMPK-related kinases proliferated and diversified to mediate the signaling of various abiotic stresses (Zu, 2016). Chromatin remodeling complexes have been found to be active during responses towards different stresses, such as *AtCHR12*, which is an SNF2/Brahma-type chromatin remodeling protein. Its paralog, *AtCHR23*, mediates growth responses under abiotic stress (Mlynárová et al., 2007; Folta et al., 2014), while SPLAYED (SWI/SNF class chromatin remodeling ATPase in *Arabidopsis*) is involved in biotic stress signaling and resistance towards pathogen (Walley et al., 2008). In the Solanaceae plants, the expression of the *SlyWRKY75* gene is induced in response to biotic stress (López-Galiano et al., 2018).

### Role of Histone Chaperones in Stress Tolerance

**Figure 2:**
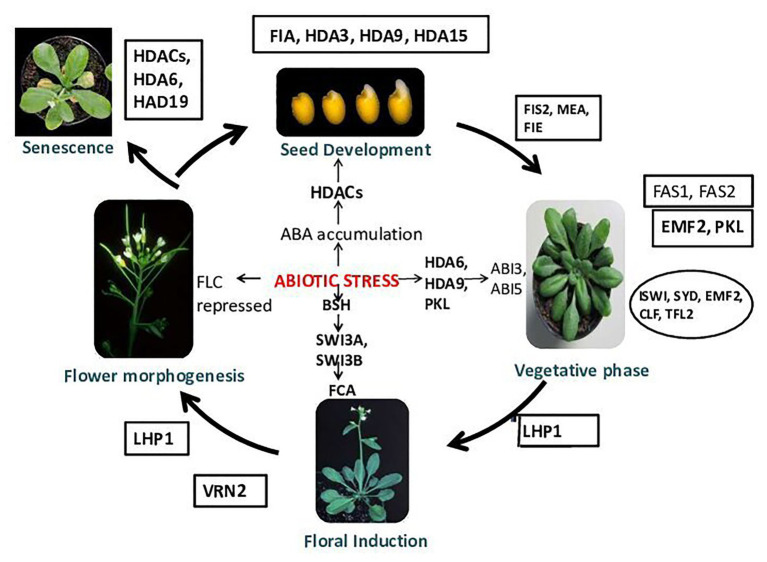
Chromatin modifications and plant development. Chromatin remodelers FIA and HDA3 play an important role during normal seed development. During stress, because of abscisic acid (ABA) accumulation, histone deacetylases (HDACs) get activated and helps in seed germination. HDA9 shows involvement in seed dormancy and germination (Baek et al., 2020). HDA15 regulates light-controlled hypocotyl elongation and regulates seed germination in the dark (Chen et al., 2020). FAS1 (FASCIATA), FAS2, EMBRYONIC FLOWER 2 (EMF2) [EMF genes repress reproductive development by delaying the vegetative-to-inflorescence (V/IF) and inflorescence-to-flower (IF/F) transitions]. The early-flowering/terminal flower phenotypes of the transgenic plants harboring the antisense EMF2 support this hypothesis. emf2-like and tfl1-like phenotypes demonstrate the role of EMF2 in the repression of the V/IF and IF/F transitions, whereas early flowering under SD conditions suggests that EMF2-mediated, photoperiod-dependent regulation of the V/IF transition, PICKLE (PKL), Imitation Switch (ISWI), SYD, fertilization-Independent Endosperm (FIE), CLF (CURLY LEAF), and TFL2 helps in normal vegetative growth. During abiotic stress, HDA6, HDA9, and PKL activates ABI3 and ABI5. VRN2 functions during floral induction. In a stressed condition, BSH (SNF5-type protein) gets activated and binds to SWI3A and SWI3B, which activates FCA. FIS2, MEA (MEDEA), and fertilization-independent endosperm (FIE) proteins operate in the same system of control of seed development. In *Arabidopsis*, the genes MEA and FIS2 encode the polycomb group (PcG) protein. The genes MEA, FIS2, and FIE repress seed development until the double fertilization event that follows pollination provides the signals for embryo and endosperm development. After fertilization, the activity of MEA, FIS2, and FIE can be detected in the endosperm tissue, and the activity of FIE activity is also found in some other sporophytic tissues (Guitton et al., 2004). LIKE HETEROCHROMATIN PROTEIN 1 (LHP1) has been proposed as a plant-specific subunit of PRC1 that could bind the H3K27me3, which is established by PRC2, and is required for a functional plant PcG system. LHP1 has been observed to control flowering time primarily by recognizing and binding to H3K27me3 and interacts with FLOWERING LOCUS T (FT) chromatin repression of FT expression (Feng and Lu, 2017). During stress, *FLC* gets repressed; in the senescence of plants, HDACs, HDA6, and HDA19 play vital roles (Wageningen Seed Lab, 2007).

Genome-wide responses, independent of transcriptional reactivation, inclusive of reduction in nucleosomal density, provide the first evidence of involvement of histone chaperones in poststress periods. In this context, mutants of CHROMATIN ASSEMBLY FACTOR 1 (CAF1; Pecinka et al., 2010; a histone chaperone complex facilitating H3 and H4 incorporation onto the neosynthesized DNA molecule) were impaired in nucleosome reassociation. FASCIATA 1 (FAS1), FASCIATA 2 (FAS2), and MULTICOPY SUPPRESSOR OF IRA 1 (MSI1) are three subunits of CAF1 (Figure 2). It is observed in *A. thaliana* that the vigor of *CAF1* mutants reduced over several generations (Kaya et al., 2001). When developmental phenotypes, transcriptomes, and DNA cytosine-methylation profiles were compared in *CAF1* mutant plants of various generations, it was seen that phenotypes related to shoot and root growth were majorly affected in successive generations of *CAF1* mutants. Limited changes in the expression of the gene were found in early and late generations of the fasciata *(fas)2-4 CAF1* mutant. The maternal participation to the phenotype severity is more than the paternal contribution when early and late generation *fas2-4* plants were crossed. It shows that the preferred maternal transmission uncovers a more prominent reprogramming of epigenetic data in the male in comparison to female germline. Epigenetic mechanisms underlie the progressive developmental phenotype aggravation in *CAF1* mutants in *Arabidopsis* (Mozgova et al., 2018).

**Figure 3:**
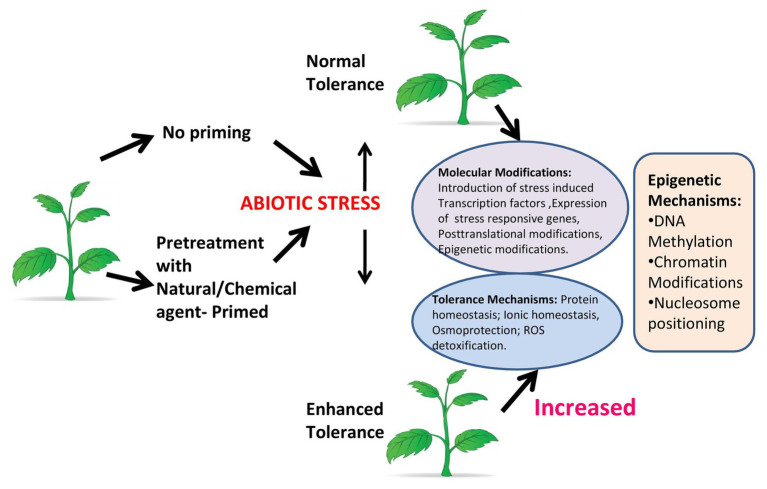
Plant priming. Under abiotic stress, a plant that is not primed shows normal tolerance, while a primed plant shows enhanced tolerance by increasing molecular functions and inducing tolerance mechanisms. Epigenetic modifications like DNA methylation, chromatin modifications, and nucleosome positioning have a major role in response to stress in primed plants.

Fasciata mutants have been reported to show pleiotropic effect in *A. thaliana*. *Arabidopsis* CAF1 is necessary for the maintenance of seedling architecture, trichome differentiation, and proper leaf size. CAF1 mutants show defects in shoot meristems. As leaf shape is primarily maintained during outgrowth of leaf primordia, the function of CAF1 is necessary for developing lateral organs and organ primordia, suggested by the strong FAS1 expression in leaf primordia (Exner et al., 2006). *fas* mutants have been observed to fail in maintaining proper expression of WUSCHEL (WUS) in SAM and SCARECROW (SCR) in RAM (Schoof et al., 2000). This shows the critical role of CAF1 in the organization of SAM and RAM during postembryonic development. In *Arabidopsis*, *fas1* and *fas2* mutants show dark green, abnormally shaped leaves, abnormal floral organs, short roots, the inability of the breakdown of meristem for distinct organs development, and thus reduced fertility (Leyser and Furner, 1992). FAS5, which is a TOP1ALPHA, a DNA topoisomerase, is not part of the CAF1 complex, and like other fasciata mutants, *fas5* mutant shows pleiotropic defects. The *fas5* mutation results in a change in the leaf and stem shape and favors the transition to the reproductive phase, leading to SAM fragmentation and tumor development on the stem. The notable increase in the SAM size in *fas5* plants in comparison with the wild-type plants suggests the role of FAS5 in WUS activity (Albert et al., 2015).

In *Arabidopsis*, MSI1 is having an important function in polycomb repressive complexes (PRC2) due to which *msi* mutants are lethal to the embryo (Köhler et al., 2003; Guitton et al., 2004 or Derkacheva et al., 2013). CAF1 plays an important role in the heterochromatin organization. It also helps in the maintenance of transcriptional gene silencing, which includes regulation of endoreduplication, homologous recombination, inactivation of certain TEs, and regulation of cell cycle duration (Mozgova et al., 2018). Stress-responsive genes mainly show progressive transgenerational upregulation in *fas2* and also affected *by* nucleosome depletion in *fas2*. These genes lack transcriptional repression in *fas1* and *fas2* mutant plants. Therefore, *Arabidopsis* CAF1 play a role in the stable repression of stress-responsive genes. CAF1 is necessary for effective resetting of stress-induced chromatin modifications, due to which it may be recommended that the enhanced stress responses along with inability to reset stress-induced chromatin states underlie the transgenerational aggravation of the CAF1 mutant phenotype. These histone chaperones are responsible for histone storage, assembly (Zhu et al., 2006), and eviction. Histone chaperones are differentially controlled in different plants under similar stress conditions (Tripathi et al., 2015). Stress-responsive genes are upregulated in mutants absent in ASF1 or CAF1 proteins and other H3/H4 chaperones (Schönrock et al., 2006; Weng et al., 2014). Plants lacking ASF1 or having the truncated NUCLEOSOME ASSEMBLY PROTEIN 1 (NAP1) and H2A-H2B chaperone (Weng et al., 2014) show hypersensitivity to stress (Chen et al., 2018a).

### Role of Histone Modifications in Stress Tolerance

Gene expression can be affected by epigenetic factors by the addition of small functional groups (methyl, acetyl, etc.) on DNA or histones (Banerjee and Roychoudhury, 2017). Methylation of DNA by DNA methyltrasferases (DNMTs) and chromomethylases (CMTs) brings about gene silencing. Histone methylation can be a positive mark of transcription if lysine 4 of histone 3 is methylated (H3K4Me1/2/3), but methylation of lysine 9 of histone 3 is a repressive mark of transcription (H3K9m2), a hallmark of constitutive heterochromatin. A similar case is reported for H3K27me1 in plants. However, H3K27me3 deposited by the polycomb pathway is a mark of “facultative” heterochromatin, involved mostly in the repression of developmentally regulated genes. Acetylation of histones by histone acetyltransferases (HATs) increases the negative charge on protein surfaces, reducing interaction with negatively charged DNA. Acetylation of histones thus results in the loosening of condensed chromatin, facilitating transcription. On the contrary, the removal of an acetyl moiety from histones by HDACs (also referred to as lysine deacetylases) facilitates condensation of chromatin (Füßl et al., 2018; Table 2). In rice, OsDSI modulates histone deacetylation to repress salt stress (Julkowska, 2018).

**Table 2:** Chromatin-associated factors and chromatin remodeling proteins.

Chromatin-associated factors and chromatin remodeling proteins	Functions	References
HAT	Transcriptional response to various biotic and abiotic stress	Stockinger et al., 2001; Vlachonasios et al., 2003
Subunit of elongator HAT complex	Phenotypes of oxidative stress tolerance, ABA hypersensitivity, and increased accumulation of anthocyanin in the mutants of four subunits	Zhou et al., 2013; Pfab et al., 2018
HDAC	Salinity stress tolerance phenotype in transgenic plants overexpressing AtHD2C	Sridha and Wu, 2006
Homolog of human TBC	Freezing stress-hypersensitive phenotype in hos15 mutants	Zhou et al., 2013
Subunit of polycomb group protein	Drought stress tolerance phenotype in cosuppression transgenic plants of MSI1	Alexandre et al., 2009; Wang and Shen, 2018
HMG protein	Phenotype of decreased seed germination rate in transgenic plants overexpressing HMGB1, phenotypes of retarded germination and subsequent growth in transgenic plants overexpressing HMGB2	Lildballe et al., 2008
ATP-dependent chromatin remodeling factor	Phenotype of growth arrest of primary buds and stems under the drought and heat stress in transgenic plants overexpressing AtCHR12, phenotype of less growth arrest under the drought and heat stress in atchr12 mutants, phenotype of reduced sensitivity to ABA-mediated inhibition of seed germination and growth in swi3b mutants	Mlynárová et al., 2007; Saez et al., 2008
CHD4	Signaling and repair after DNA damage	Larsen et al., 2010
BRM (BRAHMA)	Modulates response to ABA by preventing premature activation of stress response pathways during germination	Buszewicz et al., 2016
CHR5	Plant immune responses and nucleosome occupancy	Zou et al., 2017
CHD3	Promotion of sporophytic and gametophytic generations	Carter et al., 2016
SWI3C	Modulates gibberellin responses	Sarnowska et al., 2013

It was shown in *Arabidopsis* and rice that, upon stress, histone variants are also differentially expressed like histone chaperons (Hu et al., 2008). The H2A variant H2A.Z is downregulated under drought or salt stress in rice and *Arabidopsis* (Nguyen et al., 2017). H2A.Z has been found to be a key element for the role as a thermosensor (Kumar and Wigge, 2010) and shows the function of H2A.Z in chromatin responses during stress (Talbert and Henikoff, 2014). H2A.W found in heterochromatin is involved in decondensation induced by stress (Yelagandula et al., 2014).

Furthermore, Plants contain a distinct subclass of variants of H1 that are stress inducible (Jerzmanowski, 2007) and, when overexpressed, confer tolerance to several abiotic stresses (Wang et al., 2014). In *Arabidopsis*, H1 variants are having a major role in the molecular and spatial chromatin organization. H1 takes part in gene expression, as it is having distinct roles in euchromatin and heterochromatin (Rutowicz et al., 2019). Three variants of H1, H1.1, and H1.2 (canonical H1 proteins that are constitutively expressed), and H1.3 (involved in plant stress tolerance) are present. H1.3 is upregulated during high or low light stress conditions. H1.3 is required for both stomatal functioning under typical growth conditions and adaptive developmental responses to combat light and water deficiency. H1.3 is expressed in stomatal guard cells and can be induced by drought or stresses that signal through abscisic acid (Rutowicz et al., 2015). Plant chromatin combats stress by modulating histones by posttranslation modifications (Kim et al., 2015; Meyer, 2015). In response to stress, changes in a specific histone modification can either be global or local. Specific changes including the formation of H3K9ac (Lee et al., 2014; Widiez et al., 2014) and H3K4me3 (Ding et al., 2019) in salt or drought-responsive genes (Tardieu et al., 2018) in various plant species are responsible for stress tolerance. Abiotic stresses result in global hyperacetylation of histones in rice and maize (Fang et al., 2014; Makarevitch et al., 2015).

### Role of DNA Modifications in Stress Tolerance

DNA can also be modified by methylation in response to diverse stresses. Gene expression is maintained by the balance of methylation and demethylation at target promoters (Le et al., 2014). Modification in this equilibrium can affect the biotic stress response either negatively (Lee et al., 2014) or positively (Dowen et al., 2012). Stress conditions induce necessary changes and modifications in chromatin structure, which facilitate selective gene expression. It remains to be understood how stress signals are coordinated to drive gene activation and changes in the higher-order organization.

## Chromatin Architecture at Promoters During Plant Stress Tolerance

The promoter is an array of cis-regulatory elements that helps in the expression of the gene present downstream to it. The function of the core sequences like ACGT (Mehrotra and Mehrotra, 2010; Mehrotra et al., 2013), TGAC (Dhatterwal et al., 2019), a cis-regulatory element, and many others have revealed that cis-regulatory elements influence the gene expression either positively or negatively. Mehrotra et al. (2011) have discussed strategies to design synthetic promoter modules. Mehrotra et al. (2017) have discussed the modular nature of transcription and discussed the principles of rational combinatorial engineering; furthermore, they highlighted the importance of customized transcriptional units. A synthetic promoter is a region of DNA with a core-promoter region (or minimal promoter sequence) and multiple repeats or combinations of heterologous upstream regulatory elements (cis-motifs or TF-binding sites). Synthetic promoters are designed by the fusion of a minimal promoter to a heterologous promoter sequence at its 5' end and to a reporter gene (GUS, LUC, CAT, etc) at its 3' end (Lange et al., 2018). These synthetic constructs are introduced in plant cells by *Agrobacterium*-mediated transformation, biolistics, or, electroporation, and then, the expression of the reporter gene is studied. The core promoter region contains TATA box, which recruits RNA polymerase II, thus forming the preinitiation complex by assembling general transcription factors. The synthetic transcriptional units are the precise combination of coding and regulatory DNA sequences designed for the desired function in crop plants (Liu and Stewart, 2015). This synthetic biology is an important tool for the genetic modification of plants, thus can increase crop productivity under different environmental stresses.

SWI/SNF complexes also regulate noncoding transcription arising from promoters, enhancers, intergenic regions, and transcription termination sites (TTS) of protein-coding genes. *Arabidopsis* BRM binds to proximal promoter regions as well as the distal region of the promoter, gene bodies, and gene terminators, whereas yeast SNF2 ATPases bind specifically to promoters near the TSS site. Archacki et al. found that the binding of BRM at terminator sequences, depending on the locus, can promote or repress the transcription of antisense transcripts. Thus, it is for the effect of BRM at its gene targets that can positively or negatively regulate their transcription. In plants, SWI/SNF complex regulates promoter-centered gene function as well as controls the expression of a large number of its direct targets through their 3' ends. The regulation of noncoding RNA (ncRNA) originating from TTS by BRM does not depend on the presence of linked sense promoters, which suggests that 3'-bound BRM utilizes antisense promoters to maintain sense expression of those genes. It has been observed that the antisense transcripts arising therefrom and the TTS regions of genes have been implicated in environmental signals sensing in many systems, including cold sensing by the *FLC* 3' region and sulfur sensing by the 3' untranslated region (UTR) of SULTR2; 1 in plants, or yeast, the requirement for the 3' region of KCS1 for phosphate sensing. This suggests that a large fraction of the 3' SWI/SNF targets are stress-related genes (Archacki et al., 2017).

## Somatic Memory-Chromatin Architecture

Chromatin is broadly investigated as a major regulatory component for gene expression; it is also pertinent to investigate epigenetic mechanisms. *In vitro* somatic embryogenesis induced in response to external signals is an example of plant developmental plasticity developed by the chromatin-regulating molecular machinery (Fehér, 2015; Lämke and Bäurle, 2017). Plants show an interesting phenomenon that furthers our understanding of somatic inheritance vis-à-vis stress. It has been observed that treating plants with mild stress facilitates accelerated and enhanced responses to future challenges (Holeski et al., 2012), known as plant priming, of which chromatin is a part (Box 1). The term acquisition of thermotolerance is used when a plant is primed due to moderate heat stress (HS) and thus can tolerate high temperatures in comparison to an unadapted plant. The primed state is maintained over several days (known as maintenance of acquired thermotolerance or HS memory) after returning to normal temperatures, and this maintenance is genetically distinguished from HS priming. During HS priming, heat shock transcription factors (HSFs) get activated and increases the expression of heat shock proteins (HSPs), which then, through their chaperone activities, assist in protein homeostasis. This HS response is preserved in animals, animals, and fungi. In plants, more than 20 members of the HSP family are reported. At least eight HSFs are observed to play role in heat stress response in *Arabidopsis*. The knowledge regarding the mechanism of HS memory is not well understood. Using microarray analyses, a number of HS memory-related genes are identified, comprising genes encoding small HSPs (such as HSP21, HSP22.0, and HSP18.2) and ASCORBATE PEROXIDASE 2. The expression pattern of these genes found to be strong in the case of inducible HS when comparing with nonmemory genes (like HSP70 and HSP101). HSFA2 was reported to be the most strongly heat-induced HSF, as it is required specifically for HS memory. The first reported HS memory-associated gene, specifically involved in HS memory is HSA32. It was studied that HSA32 is required for HSP101 protein stability, which suggests a similar role to chaperons. ROF1, which is the peptidyl-prolyl-isomerase and member of the FK506-binding protein family, is also seen to be specifically required for HS memory by directly interacting with HSP90.1, which further interacts with HSFA2 (Baurle, 2016).

A priming exposure of young *Arabidopsis* plant to mild salt stress, which does not affect growth, leads to enhanced salt tolerance following a subsequent exposure. This tolerance is connected with gene and tissue-specific changes that last ~2 days (Sani et al., 2013). Higher resistance to bacterial pathogens, nonspecifically primed by various abiotic stresses, is associated with histone acetyltransferase HAC1 (Singh et al., 2014; see Box 1). Changes in H3K4 trimethylation were observed by dehydration stress priming (Ding et al., 2019) at particular “memory genes” (Crisp et al., 2016). To understand cold-induced epigenetic changes, vernalization was studied in *Arabidopsis*, which is a mechanism in plants by which they have a memory of earlier encounter of low temperature, and the plants thus flower only in favorable condition. The flower repressor FLOWERING LOCUS C (*FLC*) is silenced during vernalization by the polycomb repressive complex 2 (PRC2), which accumulates H3K27me3 at target loci (Baurle and Trindade, 2020).

Box 1Plant priming: preparing plants to tolerate future adverse conditions. Plant priming/defense priming (Martinez-Medina et al., 2016), which is also known as hardening, can be initiated in response to environmental stress [light (Han et al., 2018), temperature (Friedrich et al., 2019), water, etc.] event that acts as a cue indicating an enhanced probability of facing that specific stress factor in the future (Filippou et al., 2013). Plants enter in the primed state (PS) following perception of the cue in which the activation of the protection responses is faster and stronger when a stress pressure is encountered (Beckers and Conrath, 2007; Conrath, 2009; Ellouzi et al., 2013; Sani et al., 2013). The impact of stress exposure on the physiology and growth of primed plants can be remarkably diminished in comparison with nonprimed plants. Plants can also enter the PS by chemical priming, which involves exposure to a natural or synthetic chemical compound that acts as a priming agent (Savvides et al., 2016). Chemical priming gives opportunities for more effective use of plant priming in plant stress physiology studies and crop stress management. There are several types of molecules having the potential to act under specific conditions as a priming agent against a range of different abiotic stresses (Islam et al., 2009). A review reveals a vast range of chemical priming agents, including amino acids [e.g., proline (Li et al., 2014)] hormones [e.g., salicylic acid (Tanou et al., 2009)], reactive oxygen-nitrogen-sulfur species [RONSS (Christou et al., 2014)] and even water [i.e., hydropriming (Casenave and Toselli, 2007)]. These agents are effective in inducing plant tolerance to several individually applied abiotic stresses or biotic stresses. Primed plants show either faster and or stronger activation of the various defense responses that are induced by either pathogens or insects, or in response to abiotic stress. If the stress recurs, the benefit to the plant being primed for that particular stress response is in facilitating a more rapid response. This provides the advantage of enhanced protection without the costs associated with constitutive expression of stress related genes (Figure 3).

## Intergenerational and Transgenerational Stress Memory

**Figure 4:**
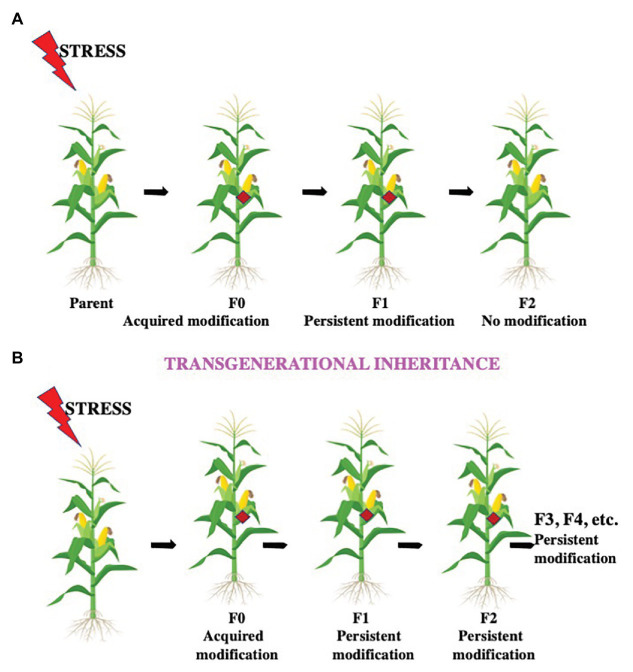
Intergenerational and transgenerational inheritance. Environmental conditions like heat stress, temperature fluctuations, light duration and intensity, insect attack, osmotic imbalance, etc. can influence future generations by different modes. **(A)** The offspring after these conditions with the altered epigenetic structure is the F0 generation. If the modification is successfully passed on from the F0 generation only to their offspring, the F1 generation, the modification is termed as an intergenerational inheritance. **(B)** If the modification is successfully transferred from the F1 generation to the F2 generation and further generations, the change is termed as transgenerational inheritance.

Lamarck in the nineteenth century first hypothesized that traits acquired during an organism’s life could be transmitted from one generation to the next generation, which is known as Lamarckism (Lamarck’s theory) or the theory of “inheritance of acquired characteristics.” According to Lamarck, alterations in phenotypic traits are a result of the environment and are associated with evolution. Lamarckism says that simple organisms tend to evolve into more complex ones by an adaptive force. The environment creates needs to which organisms respond by utilizing features, which are then emphasized or weakened through use and disuse; this generates characteristics that an individual organism acquires and then are pass on to its offspring. Plants have elaborate mechanisms to deal with different environmental conditions. When the memory effect is present only in the first stress-free generation, it is called intergenerational memory, while if the memory is traceable in a minimum of two stress-free generations, it is termed transgenerational memory (Tardieu et al., 2018; Figure 4). Transgenerational memory (TSM) likely consists of an epigenetic basis, i.e., the phenotypic traits possessed by the offspring are a result of environmental stimulus in an earlier generation but not in the parent or offspring. There are reports showing that there is an increase in somatic homologous recombination (SHR) in the parental generation when treated with the flg22 elicitor or UV-C irradiation that indicates the presence of a stress-induced transgenerational memory (Molinier et al., 2006), which remained elevated during numerous unstressed generations, showing an epigenetic basis (Kinoshita and Seki, 2014). During transgenerational memory, the DNA methylome is relatively unaffected by stress-induced changes in *Arabidopsis* (Ganguly et al., 2017). According to some studies, hyperosmotic stress priming will develop when plants were subjected to stress during their vegetative development for at least two generations (Pecinka et al., 2009; Murgia et al., 2015). The maternal parent is likely responsible for this intergenerational stress memory. It is suggested that, in the male gametes, DNA glycosylase DEMETER (DME) inhibits paternal inheritance, and it is restored in *dme* mutants (Choi et al., 2002). DME encodes a protein having DNA glycosylase and nuclear localization domains, and it is expressed mainly in the central cell of the female gametophyte, the progenitor of the endosperm. DME is involved in the demethylation of transposable elements (TEs) and repetitive sequences, which lead to TE upregulation and small interefering RNA (siRNA) production in endosperm and vegetative cells (Saze et al., 2012). The role of DME is also studied in genomic imprinting. Using base excision repair mechanism, DME can excise methylated cytosine bases from any sequence, which is similar to *A. thaliana* glycosylases DEMETER LIKE 2-3 (DML2-3) and REPRESSOR OF SILENCING 1 (ROS1; Ortega-Galisteo et al., 2008; Gehring et al., 2009). DME demethylating repetitive sequences, TEs, and targeted regions seem to be partially identical in the central cell and the vegetative nucleus, as it is active in both the central cell of the female gametophyte as well as the vegetative cell of pollen (Park et al., 2016). It has been proposed that the demethylation of TEs in the central cell and the vegetative cell is part of a defense mechanism so that these TEs can be silenced in the egg and sperm cells (Calarco et al., 2012; Ibarra et al., 2012). Due to the demethylation of TEs, transcriptional activation gets promoted, and thus, production of siRNAs takes place (Slotkin et al., 2009). These siRNAs can then promote DNA methylation *via* the noncanonical RNA-directed DNA methylation (RdDM) pathway, which uses them as guides and target the DNA methylation machinery to homologous sequences (Cuerda-Gil and Slotkin, 2016; Zhang et al., 2018). It is hypothesized that the siRNAs that are produced in the central cell and vegetative nucleus travel to the adjacent gametes (the egg and sperm cells) and initiate DNA methylation of TE sequences there, resulting in their silencing (Calarco et al., 2012). As imprinted genes are often found to be enriched in TEs in their flanking regions, DME-mediated methylation of these TEs may affect the expression of neighbor-imprinted genes (Hatorangan et al., 2016; Yuan et al., 2017). The role of RdDM in initiating methylation of the paternal alleles of some MEGs and the activity of DNA METHYLTRANSFERASE 1 (MET1) and CHROMOMETHYLASE 3 (CMT3) are needed for the CG and CHG methylation levels maintenance in sperm cells, leading to epigenetic inheritance (Calarco et al., 2012). DNA methylation and H3K27me3, with some additional epigenetic modifications such as H3K9me2, are recognized to be responsible for the imprinting of some genes (Batista and Kohler, 2020). These variations occur for the transcriptional regulation of abiotic stress genes in plants. As these changes in the epigenome are stably inherited and passed to further generations, knowledge about these changes is crucial for stress management in plants. The knowledge of specific epigenetic marks with particular stressors would permit the generation of stress-tolerant plants by identification of the above-mentioned techniques.

RNA interference plays a critical role in epigenetic modification of histones and DNA, as it can repress target genes at the transcriptional level. Constitutive heterochromatin is a major source of siRNAs involved in the silencing of transposable elements. siRNAs are required for maintenance of asymmetric DNA methylation (CHH context) following mitosis and meiosis to ensure epigenetic inheritance (Law and Jacobsen, 2010). Double-stranded RNA (dsRNA), which can increase posttranscriptional silencing of cognate genes, gets cleaved by the RNase III enzyme, Dicer into siRNAs. These siRNAs guide the target effector complexes, such as the RNA-induced silencing complex (RISC), to endogenous transcripts leading to degradation or translational inhibition. These findings suggested a preserved nuclear role for RNAi in transcriptional gene silencing (TGS). As it occurs in the germline, TGS can lead to transgenerational inheritance. In *Schizosaccharomyces pombe*, a role for RNAi in TGS was observed where it is necessary for the formation of constitutive heterochromatin at pericentromeric. The release of the passenger strand from *Ago1* and dsRNA requires catalytic activity and thus is necessary for the pairing of bases between loaded siRNA and their targets. These interactions provide the RNA-induced transcriptional silencing complex (RITSC) a critical place, integrating transcription and chromatin modification, which creates a positive loop between siRNA generation, RITSC localization, and H3K9 methylation. In *S. pombe*, the coupling of transcription, production of siRNA, and silencing indicates that TGS occurs in cis; however, in plants, it has been seen that it can also occur in trans. In *A. thaliana* microRNA (miRNA)-directed siRNA biogenesis is a mechanism that particularly targets transposon transcripts and triggers epigenetic reactivation during reprogramming of the germ line (Creasey et al., 2014). In *Arabidopsis*, hetrochromatin is majorly defined by transposable elements and related tandem repeats, under the effect of the chromatin remodeling ATPase DDM1. siRNA possesses these sequences, indicating a role in guiding DDM1. The regulation of the euchromatic, imprinted gene *FWA*, as its promoter is hence can be understood by DDM1 and the DNA methyltransferase MET1, as they provide the transposable-element-derived tandem repeats that are associated with siRNAs (Lippman et al., 2004). Analysis of small RNA profiles and DNA methylation profiles identifies regions regulated by miRNA/siRNA-mediated DNA methylation, which involves the epigenetic inheritance of stress effects. Thus, the role of miRNA and siRNA in biotic and abiotic stresses in plants can be understood. The knowledge of small RNA-guided stress regulatory networks provides new insights for genetically improved plant stress tolerance. Manipulation of miRNA or siRNA-guided gene regulation can be used to engineer stress resistance in plants.

During cellular proliferation, the stable inheritance of epigenetic modifications is necessary to maintain cell identity. In plants, the transmission of H3K27me3-silenced state requires the replication-dependent histone variants H3.1 (Jiang and Berger, 2017). This H3.1 provides PRC2 function, managing proper maintenance of H3K27me3 domains and ensuring the silencing of developmental genes. In *A. thaliana*, flowering is initiated when H3K27me3 established at the floral repressor FLOWERING LOCUS C (*FLC*), which is a result of H3.1 deposition during DNA replication. At *FLC*, H3K27me3-mediated silencing finally reset in the future generation to ensure transcriptional reactivation in the early embryo (Tao et al., 2017).

The involvement of active demethylation in the loss of H3K27me3 has been suggested as implicated in the epigenetic resetting of *FLC*. The Jumonji-C family (JMJ) histone demethylases counteracts the activity of PRC2. There are three closely related JMJ H3K27 demethylases reported were EARLY FLOWERING 6 (ELF6), RELATIVE OF ELF6 (REF6), and JUMONJI 13 (JMJ13), and all are expressed in the sperm. As PRC2 is absent in the sperm, the H3K27me3 demethylation by JMJ proteins is supposed to occur globally, whereas in somatic tissues, H3K27 demethylases occupy the border of H3K27me3 domains in presence of PRC2 (Yan et al., 2018). ELF6 and REF6 play important roles in H3K27me3 and H3K27me1 homeostasis (Antunez-Sanchez et al., 2020). *elf6 ref6 jmj13* mutant showed elevated levels of H3K27me3 in the sperm when compared to wild type, suggesting the role of active demethylation by JMJ proteins in contribution to paternal H3K27me3 resetting. These JMJ proteins are found to demethylate the di-and trimethyl H3K27 but not H3K27me1 (Song et al., 2015; Zheng et al., 2019). JMJ demethylate H3K27me3 retained upon H3.10 depletion and convert it to H3K27me1 in the sperm. The *HTR10* encodes the sperm-specific histone variant H3.10 and indicates an increased level of H3K27me1, which is unlikely a result of mono-methylation by ATXR5/6, as its only substrate H3.1 is not expressed in wild type and *htr* sperm. In quadruple *elf6 ref6 jmj13 htr10* mutant sperm, a reduction in H3K27me1 levels was observed while the level of H3K27me3 was increased, suggesting that the deposition of H3.10 replaces a prominent region of H3K27me3-marked nucleosomes and H3K27me1-marked nucleosomes formed by the action of JMJ demethylases (Borg et al., 2020). During sexual reproduction, it has been seen that these chromatin marks are failed to reset, which leads to transgenerational inheritance of histone marks, resulting in loss of DNA methylation and transposon activation. Hence, in plants, JMJ-type histone demethylases help in maintaining transcriptional states through development as well as safeguard genome integrity during sexual reproduction (Borg et al., 2020).

The intergenerational memory is mediated by DNA demethylation and RNA-mediated DNA methylation pathways in case of hyperosmotic stress (Wibowo et al., 2016). Genome-wide methylation analysis helped in the identification of differentially methylated regions (DMRs) linked with this intergenerational memory (Ferreira et al., 2019). The promoter of the gene related to stress has two such DMRs involved in priming effect on gene expression (Wibowo et al., 2016). There are reports showing the role of intergenerational and transgenerational stress memory in biotic stresses as well (Pieterse et al., 2012; Espinas et al., 2016). (Luna et al., 2012) showed that intergenerational or transgenerational memory is evidenced by increased salicylic-acid-related defense gene induction and susceptibility to biotrophic pathogens (Slaughter et al., 2012). From there, it is suggested that, for environmental challenges that plants may encounter in their life, they prime their offspring. It has been reported that, in the extremely challenging environmental conditions of a typical *Arabidopsis* habitat, transgenerational inheritance of priming may be disadvantageous over more than one generation (Luna et al., 2012; Iwasaki and Paszkowski, 2014). A full understanding of how TSM is related to seed germination and development under environmental changes could be important in research related to stress adaptation in plants and thus could help in the selection of stress-adapted genotypes.

## Chromatin Modifications and Plant Development Under Stress

In eukaryotic cells, cellular changes and gene expression are regulated by gene regulatory mechanisms in numerous biological processes, like a response to extracellular signals, recombination, developmental reprogramming, and genome stability (Zhu et al., 2013). Changes in DNA methylation, histone variants, and histone N-tail modifications, which are induced by stress, regulate plant development under stress and stress-responsive gene expression. Control of gene expression like this in response to endogenous and environmental stimuli in plants controlled by chromatin modifications is crucial for reproductive success and proper development (Archacki et al., 2013; Efroni et al., 2013; Sarnowska et al., 2013; Qin et al., 2014; Vercruyssen et al., 2014). A drastic change is triggered in seedling morphology when it first emerges from the soil due to rapid changes in histone modifications and gene expression including growth cessation of hypocotyls, the opening of apical hook and cotyledons, and the development of chloroplasts due to its encounter to light, which is known as photomorphogenesis. The physiology, morphology, and development of the plant thus depend on the duration and quality of light as well as the presence of competitors, which can alter the amount of light reaching the plant (Perrella et al., 2020).

The embryonic and postembryonic phases are two phases of the plant developmental cycle (Chen et al., 2018b). The postembryonic phase includes the growth of the leaf, stem, and flower meristems (Ojolo et al., 2018). The uniformity of seed germination and seedling establishment gets decreased during osmotic stress. Abscisic acid (ABA) accumulation induces several HDACs in *Arabidopsis* during seed development. *Arabidopsis* HDA6 and HDA19 have crucial roles in abiotic stress signaling through the formation of repressive complexes. HDA6 regulates the function of abiotic-stress-responsive genes (ABI1, ABI2, and ERF4) by interacting with HD2C (Luo et al., 2012b), whereas HDA19 with ERF3, ERF4, ERF7, SIN3, and SAP18 are part of chromatin remodeling complexes in abiotic stress responses. The mechanism of HDA9 function in signal transduction during abiotic stress responses is little known. A model is proposed for understanding the function of HDA9 in ABA-dependent drought stress signaling in plants (Fujita et al., 2005; Baek et al., 2020). During seed germination and plant development in wild-type plants, it was observed that, to regulate ABA homeostasis, the expression of ABA catabolism-related genes (CYP707As) changed ABA from an active to an inactive form (8′-hydroxyl ABA). Whereas, in the case of drought-stress-exposed plants, HDA9 and ABI4 together function in inhibiting the expression of CYP707As. HDA9 in association with an ABA-related transcription factor functions in inhibiting gene expression by histone deacetylation. In the drought stress response of plants, HDA9 is a crucial negative regulator in transcriptional regulation of ABA-catabolism-related genes like CYP707A1 and CYP707A2. HDA9 also plays an important role in seed dormancy and stomatal closure (Figure 2). It was observed that, in the case of *hda9* mutants, seed germination was significantly increased in comparison to wild type when exposed to exogenous ABA. In the presence of ABA, *hda9* mutants showed a significantly higher percentage of fully opened green cotyledons than the wild type. To suppress the effect of negative regulators of early ABA signaling, the MYB96 transcription factor associates with the histone modifier HDA15. This MYB96 TF is known as a master transcriptional regulator that mediates several plant responses to ABA, for example, seed germination, stomatal conductance, drought tolerance, anthocyanin accumulation, hormone biosynthesis, lateral root development, and cuticular wax biosynthesis. The MYB96-HDA15 complex formed interacts with the promoters of a subset of RHO GTPASE OF PLANTS (ROP) genes (ROP6, ROP10, and ROP11) and removes acetyl groups of histone H3 and H4 from the cognate regions, thus represses their expression specifically when ABA is present. A reduction in ABA sensitivity is observed in HDA15-deficient mutants, thus are affected by drought stress. Various transcription factors from bZIP, MYC, NAC, and MYB families then get activated and initiate downstream ABA responses. HDA15 represses ROP genes and acts as a positive regulator of ABA signaling by repressing (Lemichez et al., 2001). Transgenic plants overexpressing HDA15 and MYB96 possess hypersensitivity to ABA, whereas *hda15* and *myb96* mutants show reduced ABA sensitivity (Lee and Seo, 2019). Drought stress tolerance in plants as an effect of ABA was additionally affected by HDA15. During seed germination and drought tolerance, MYB96 and HDA15 act synergistically to confer ABA sensitivity (Lee and Seo, 2019). The *HDA15* gene is observed to be induced by ABA treatment. HDA15 expression enhanced under various abiotic stress factors (mainly osmotic, cold stress). HDA15 activity also affects the expression of some ABA-responsive genes. The expression of PKL (SWI/SNF type chromatin remodeling factor) is induced by seed imbibition in *Arabidopsis*, and it mediates the repression of embryonic traits during germination. Seed germination is mediated by induction of ABI3 and ABI5 transcription factors expression in response to induced expression of PKL. This discussion suggests that there is a change in expression or activity of HDACs due to ABA accumulation, which in turn regulates growth under stress (Figure 2).

Plant reproduction includes flowering and seed development. Flowering is an essential part of the reproductive process as well as a critical developmental stage that can be susceptible to environmental stresses in plants (Kazan and Lyons, 2015). In appropriate environmental conditions, plants have mechanisms to flower. In *Arabidopsis*, during vernalization, low-temperature epigenetic mechanisms get induced, which repress the FLOWERING LOCUS C (*FLC*, a MADS-box protein) gene, which remains until progression to flowering. Due to prolonged cold, COOLAIR, which is a set of long noncoding RNA (lncRNA)-transcribed antisense from *FLC* in *A. thaliana*, gets induced, which is a characteristic of polycomb silencing. As discussed earlier in the review, the polycomb group (PcG) proteins are responsible for gene silencing in higher eukaryotes. PcG regulates many genes and several developmental processes. It has been found during cold conditions that the expression of *FLC* gets reduced when COOLAIR gets associated with the *FLC* locus. The synchronized replacement of H3K36 methylation with H3K27me3 gets disturbed at the *FLC* nucleation site when COOLAIR is removed during cold stress (Zeng et al., 2019). The role of COOLAIR in natural variation can be suggested by the slow repression of *FLC* in the slow vernalizing accession Var2-6 because of splicing of distally polyadenylated COOLAIR (Li et al., 2015). Two more lncRNAs, COLDWRAP and COLDAIR, are found to be responsible for the stable silencing of *FLC* by recruiting PHD-PRC2 to a specific chromatin region (Kim et al., 2017). The sequence similarity between lncRNAs across different plant species is not significant, while it has been found that they are positionally conserved. PRC2 is a very important complex in the developmental transition to flowering, which also takes part in several developmental processes in plants. In *Arabidopsis*, for PRC2-mediated H3K27me3, HISTONE DEACETYLASE 9 (HDA9)-mediated H3K27 deacetylation is necessary (Qian et al., 2012). The knowledge of COOLAIR could provide scope for understanding the mechanism of thermosensing during vernalization. LncRNAs acts as a guide for protein complexes mediating epigenetic regulation. Chromatin-associated lncRNAs maintains chromatin conformation. As lncRNAs are mobile and long, they function as bridges to mediate chromatin looping and also helps in inter- or intrachromosomal interactions. RNA hybridizes with DNA and form R-loops contributing to gene regulation. Liquid-liquid phase separation is also mediated by RNA, as it can act as a multivalent scaffold for the binding of RBPs. The role of lncRNAs in several gene regulatory networks associated with various biological processes like plant development and stress responses is studied. A few lncRNAs have been found to perform targeting functions by chromatin modification complexes, coactivation or cosuppression of trans-acting RNAs.

In *Arabidopsis*, FCA and FPA proteins downregulate flowering repressor *FLC* and form an autonomous flowering pathway. DNA methylation can be regulated by both FCA and FPA, which are RNA-binding proteins (Bäurle et al., 2007). In the compartments without membrane, the concentration of proteins and nucleic acids is a very crucial part of cellular biochemistry. The formation of these biomolecules takes place by measures including liquid-liquid phase separation, as the interactions between different multivalent macromolecules generate clear liquid-liquid-demixing phase separations, creating micrometer-sized liquid droplets in an aqueous solution. FCA involves in phase separation, as it possesses prion-like domains that phase separated *in vitro* and shows behavior *in vivo*. The construction of FCA nuclear bodies requires a coiled protein, FLL2, which enhances the proximal polyadenylation of FCA. In the *Arabidopsis* genome, this proximal polyadenylation decreases transcriptional read through (Li et al., 2012). The expression of these FLL2 has been seen to increase the number and size of FCA nuclear bodies. To increase polyadenylation at specific sites, FCA nuclear bodies compartmentalize 3'-end processing factors. It is observed that coiled-coil proteins can promote liquid-liquid phase separation (Fang et al., 2019). FCA is considered as a part of the signaling pathways mediating plant adaptation responses to high temperatures (Lee et al., 2015). FCA RNA-binding protein act as a transcriptional regulator through modifying RNA processing or chromatin modification. Various enzymes and regulators associated with the transcriptional and posttranscriptional control of plant reactions to environmental signals are mediated by FCA. FCA generally works in these processes by RNA metabolism and chromatin alteration.

The expression of PsSNF5, which is a chromatin remodeling gene, is induced by drought stress (*Pisum sativum* SNF5). PsSNF5 interacts with *Arabidopsis* SWI3-like proteins (SWI3A and SWI3B), which further interacts with FCA (Rios et al., 2007; Figure 2). Flowering time and stress responses are regulated by ABA-induced SNF5 and FCA by chromatin remodeling. Premature leaf senescence due to abiotic stresses leads to reduced photosynthesis. Jasmonic acid and ethylene-responsive-HDACs, HDA6 and HAD19 (Wu et al., 2008), alter leaf senescence, while HDA19 antisense transgenic plants/T-DNA mutants showed early senescence (Zhou et al., 2005; Ay et al., 2014; Figure 2).

## Effect of Stress on Chromocenters

Chromocenters are dense heterochromatic regions, heavily packed with DNA and proteins present in the nucleus of some cells. Emil Heitz (1928) historically identified heterochromatin as the nuclear material that remains highly condensed within the interphase nucleus. He named these regions “heterochromatin” to distinguish them from the regions showing variable staining and condensation, which he called “euchromatin.” The functional properties and composition of chromatin structure came into the picture very late; however, the distinction between heterochromatin and euchromatin was provided many years back (Passarge, 1979). A major point of discussion comes from the structure of heterochromatin, which is cytologically visible upon different types of stresses within *Arabidopsis* nuclei. At a specific developmental stage or particular environmental condition, these chromocenters can be transiently decondensed. It is proposed that nuclear organization modifications and stress responses have a functional connection (Groves et al., 2018). Stress can be accompanied by dramatic morphological alterations in the organization of plant nucleoli and the protein content. These changes are presumably related to alterations in diverse nucleolar transcriptional activity under stress conditions (Kalinina et al., 2018). The chromocenters are enriched in transposable elements, transcriptionally silent 45S and 5S rDNA arrays, and centromeric and pericentromeric satellites, which can be seen clearly in *Arabidopsis* nuclei at interphase (Fransz and Jong, 2011; Benoit et al., 2013). The formation of euchromatic loops from chromocenters has been visualized by DNA fluorescence *in situ* hybridization (FISH) experiments and more recent Hi-C analysis, revealing their role in the spatial organization of chromosomes (Ron et al., 2013; Feng et al., 2014). Thus, chromocenter organization has been extensively utilized to understand chromatin modifications under stress or during development in *Arabidopsis* (Benoit et al., 2013). Interestingly, the temporary decondensation of chromocenters that happens during the floral transition occurs in terminally differentiated leaf tissue (Tessadori et al., 2007), and it is still unclear whether it occurs in the meristem as well.

At the time of seed germination and maturation, the alteration of chromocenter structure also takes place in the nuclei of the cotyledon (Zanten et al., 2012) and postgermination development (Mathieu et al., 2003; Douet et al., 2008). Chromatin modifications are related to process linked to the development of the plant as well as external stress signals, like temperature-stress-induced dedifferentiation (van Dam, 2014), lightly shape nuclear architecture (Bourbousse et al., 2015) and gene expression (Kaiserli et al., 2018), and reprogramming of microspores. There are some reports showing how the nuclear structure is affected by abiotic stresses unrelated to specific developmental processes in the rye and rice seedlings, in which upon heat stress, the 45S rDNA (Santos et al., 2011) loci undergo decondensation. In *Arabidopsis*, it was found that the stem cell expression is mainly dependent on the developmental stage but also contain a core set of stem-cell-specific genes, some of these genes are involved in epigenetic silencing. In meristems before flower induction, increased expression of transposable elements correlates with enhanced CHG methylation during development and reduced CHH methylation, before stem cells enter the reproductive lineage (Sasaki et al., 2019). This shows the occurrence of epigenetic reprogramming at an early stage and its role in genome protection in stem cells during germline development (Gutzat et al., 2020). In the *Arabidopsis* leaf tissue, after prolonged heat stress, centromeric repeats and 5S rDNA decondensation occur (Pecinka et al., 2010). In *Arabidopsis*, HEAT INTOLERANT 4 (HIT4) was discovered for heat-stress-intolerant mutants; in excessive heat stress, it is required for chromocenter decondensation upon heat stress (Wang et al., 2013, 2015).

## Plant Response to Stress: the Chromatin Perspective

Plants cannot escape the myriad of biotic and abiotic stresses to which they are exposed during their life cycle. The information available highlights those changes in chromatin features; particularly, histone modifications are a key feature in plant response and adaptation to environmental insults. According to a review by Dogan and Liu (2018) and Silveira (2018), it is expected that, in the near future, there will be a wave of datasets focusing on plant epigenomes and transcriptomes in the 3D context, serving as an essential component in finding key regulators of plant chromatin folding and positioning (especially for crop plants). Changes in temperature induce specific responses modifying chromatin configurations as reported for cold (Kim et al., 2010; Roy et al., 2014) and heat (Christina et al., 2010; Kumar and Wigge, 2010; Pecinka et al., 2010) stress in higher plants and algae (Schroda et al., 2001; Lee et al., 2014). Due to global warming, guarding plants against decline due to heat stress and temperature fluctuations is becoming increasingly important (Ohama et al., 2017). Small RNAs and epigenetic regulation are involved in transcriptional regulation and heat stress memory (Kapazoglou et al., 2017). Drought signaled through abscisic acid is an extreme condition for plants and is also linked to chromatin modifications (Mehrotra et al., 2014). Experiments performed in *Coffea canephora* verified that transcriptional memory alters drought-responsive gene expression (Guedes et al., 2018). Osmotic stress or salinity is frequently associated with responses at the chromatin level. Light deficiency affects chromatin structure, signaled by light perception factors (Zanten et al., 2010, 2012). Plants exposed to chemically induced DNA damage force chromatin modifications (Braszewska-Zalewska et al., 2013; Rosa et al., 2013). Chromatin structure is also disturbed by toxic components as demonstrated by the study on seawater algae with respect to cadmium (Greco et al., 2012). In addition to these abiotic factors, it has been observed that the pathogen challenge is signaled to chromatin to induce defense gene expression (Berr et al., 2012; Schenke et al., 2014). Eventually, intrinsic responses to senescence or wounding (Gnatowska et al., 2014) can modify chromatin configurations.

Autotrophs like plants possess an impressive degree of metabolic flexibility to sense and survive under different stress conditions. Knowledge in chromatin architecture and associated modifications is important to understand varied pathways through which plants adapt themselves to various stress conditions. Chromatin organization and epigenetic modification, which can be altered by developmental or environmental stimuli, are dynamic in nature and provides a means to stabilize and condense DNA. Chromatin architecture is modulated to cope with various stresses that plants may experience. Numerous transcription factors, transcriptional memory, and small noncoding RNAs contribute towards gene expression modulation during plant stress responses (Avramova, 2015). The rearrangement of chromatin between transcriptionally inactive to transcriptionally active state facilitates access of transcription factors or other DNA binding proteins to regulate gene expression.

Stress can induce transcriptional activation as well as transcriptional repression. To bring repression of transcription, transcriptional repressor proteins counteract the activity of positively acting transcription factors. In addition, transcriptional repression is often linked with chromatin reorganization. Numerous transcriptional repressor proteins communicate either directly or indirectly with proteins that remodel chromatin or would themselves be able to impact chromatin structure. Transcriptional repression may also display “memory” of the prior transcriptionally inactive state, which is known as transcriptional repression memory (TREM). A study conducted in yeast shows that transcriptional repression of ∼540 genes occurs at a faster rate if, during carbon source shifts, the genes have been previously repressed (Gaston and Jayaraman, 2003).

Various biochemical changes take place in chromatin structure to maintain gene activity: Some of these modifications have the capacity to be stably transmitted through cell division stages, which suggest that modifications in the chromatin state could help in coping with different biotic and abiotic stresses (Gallusci et al., 2017). Further studies may help to validate the transmission of stress-induced changes in chromatin. The information can be used to increase crop yield and thus improve agricultural systems. This information can be utilized to find out the significance of chromatin remodeling proteins in regulating transcription at each step, i.e., initiation, elongation, and termination.

## Outstanding Questions

- What are the kinetics of changes in histone modifications and transcripts following the stress signal perception?
- Which transcription factor interact with which coactivator or corepressors under a given stress situation and cell type?
- What is the role of cell type in determining transcriptional regulation through its chromatin status?
- What is the exact composition of native chromatin modifying complexes in different tissues, developmental stages, and stress situations?
- Can we design epigenetic switches to regulate agronomically important traits under stress conditions?
- Can we exploit the strength of epigenome modification in horticultural crops since their breeding is difficult? Can grafting change methylation and acetylation state in horticultural crops?

## Author Contributions

SB and SM: writing and reviewing. MB and GL: reviewing and editing. RM: conceptualizing, reviewing, writing, and editing. All authors contributed to the article and approved the submitted version.

### Conflict of Interest

The authors declare that the research was conducted in the absence of any commercial or financial relationships that could be construed as a potential conflict of interest.
